# Effect of Corticosterone on Gene Expression in the Context of Global Hippocampal Transcription

**DOI:** 10.3390/ijms26104889

**Published:** 2025-05-21

**Authors:** Grzegorz R. Juszczak, Adrian M. Stankiewicz, Rafał R. Starzyński, Magdalena Ogłuszka, Aneta Jaszczyk

**Affiliations:** 1 Department of Animal Behavior and Welfare, Institute of Genetics and Animal Biotechnology, Polish Academy of Sciences, 05-552 Jastrzebiec, Poland; a.jaszczyk@igbzpan.pl; 2 Department of Molecular Biology, Institute of Genetics and Animal Biotechnology, Polish Academy of Sciences, 05-552 Jastrzebiec, Poland; 3 Faculty of Medicine, Collegium Medicum, Cardinal Stefan Wyszyński University in Warsaw, 01-938 Warsaw, Poland; 4 Department of Genomics, Institute of Genetics and Animal Biotechnology, Polish Academy of Sciences, 05-552 Jastrzebiec, Poland

**Keywords:** corticosterone, glucocorticoids, brain, transcriptome, gene expression, RNA-seq

## Abstract

The composition of genomic mediators of glucocorticoid actions in the brain remains elusive because of low-statistical-power experiments and the associated transcriptomic data with very low consistency. The problem is further exaggerated by the underrepresentation of chronic experiments and the interpretation of differentially expressed genes without understanding their contribution to the total transcriptomic activity. To fill existing gaps in knowledge, we have performed a large transcriptomic experiment, testing the effects of prolonged treatment with corticosterone on the hippocampal transcriptome (RNA sequencing). The experiment showed that prolonged treatment with corticosterone induced a set of transcriptomic effects that were replicable across treatment durations, including genes relevant for human PTSD (*Opalin*, *Pllp*, *Ttyh2*, *Lpar1*) and prolonged stress in animals (*Cnp*, *Fam163a*, *Fcrls*, *Tmem125*). Some of the affected genes are specific for oligodendrocytes, neurons, astrocytes, immune cells, the vascular system, and brain ventricles, indicating that glucocorticoids may affect all central nervous system components. The data also showed that the largest changes in expression of corticosterone-responsive genes are restricted to genes with a relatively low expression level and small contribution to the overall pool of mRNAs in the hippocampus. As a result, even a large change in the number of affected genes leads to a small change in the number of newly synthesized mRNA copies. This means, in turn, that the transcriptomic changes induced by corticosterone have low-cost effects on the brain. This specificity of transcriptomic responses also poses a challenge for the interpretation of data and constitutes a potential source of reporting bias in past studies. Therefore, there is a need for further research on products of gene expression, both at the transcriptomic and proteomic levels, during stress conditions.

## 1. Introduction

Glucocorticoids are well known to cause effects mediated by genomic mechanisms requiring altered gene expression, as indicated by early experiments performed in hepatoma cell culture [1]. However, despite several decades of research and the introduction of large-scale transcriptomic methods [2], the composition of genomic mediators of glucocorticoids in the nervous system remains elusive [3]. In fact, available data from experiments testing glucocorticoids implicated almost 10,000 genes, but very few of them have been confirmed by independent studies [3]. Such a high variability of transcriptomic data results from many factors, including technical limitations associated with past experiments, selective publishing of results, very small sample sizes typical for transcriptomic studies, and associated high rates of false positive and negative findings [4]. This problem can be solved, however, by using two complementary approaches. The first is the usage of larger sample sizes to increase the statistical power of new experiments [5,6,7] performed with more advanced technologies. The second solution is an analysis of aggregated data derived from different studies [3,4], but this approach depends on the availability of data, and previous efforts have been hampered by the underrepresentation of transcriptomic data obtained after treatments longer than 4–6 h [3]. Finally, the general problem with available transcriptomic studies is that they focus on a relatively small number of significant genes that are selectively published and interpreted, without understanding the structure of the entire transcriptome. This approach restricts our understanding of genomic mechanisms and may lead to misinterpretation of results. For example, it is common that genes displaying the largest fold changes between groups receive priority in data analysis [8,9]. However, these genes can be expressed at low levels, indicating either residual overall expression or expression restricted to a very small number of cells belonging to the investigated tissue or even derived from the blood [10]. As a result, such an approach can easily bias the conclusion towards genes with low functional significance in the investigated tissue.

To fill the existing knowledge gaps, we performed a large-sample transcriptomic experiment (48 mice in total) with prolonged corticosterone treatment ranging from 5 to 28 days to enable assessment of the replicability of findings and identification of time-dependent processes. Furthermore, we performed a global assessment of the hippocampal transcriptome to enable a better understanding of the effects induced by glucocorticoids.

## 2. Results

### 2.1. Effect of Treatment with Corticosterone on Blood Level of Corticosterone and Weight of Internal Organs

**Figure 1:**
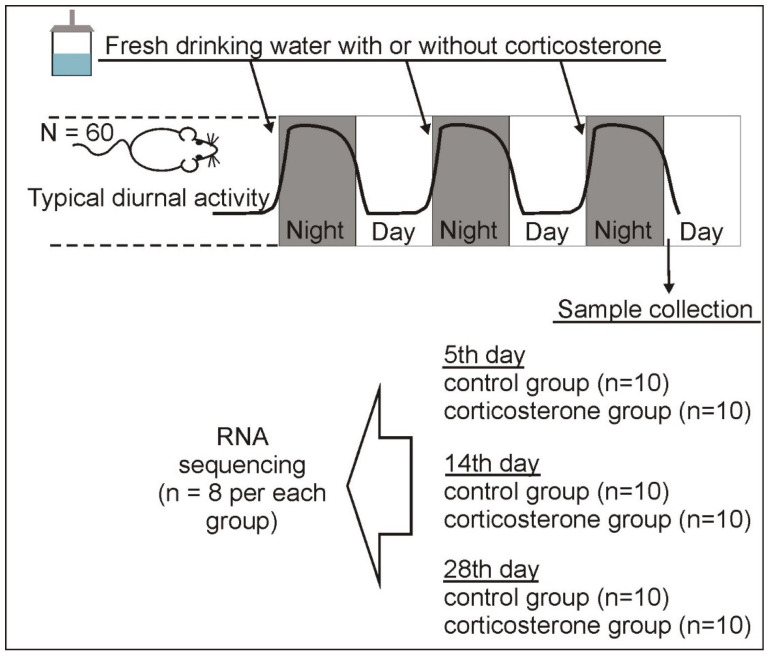
Experimental design. The treatment was preceded by a three-week period of habituation. More details in Section 4.1, Section 4.2 and Section 4.4.

**Figure 2:**
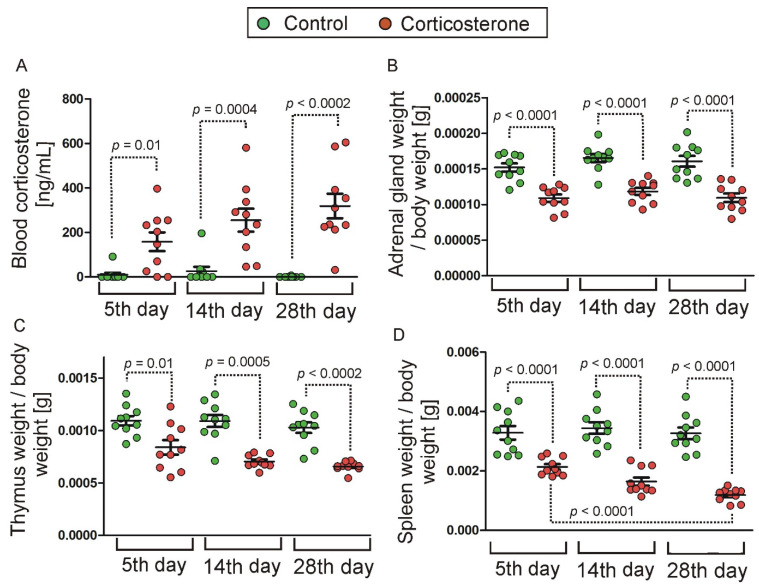
Control parameters confirming the effectiveness of the treatment with corticosterone. (**A**) Blood corticosterone. (**B**) Weight of adrenal glands. (**C**) Weight of thymus. (**D**) Weight of spleen. The *p*-values were determined with a nonparametric Mann–Whitney U test (blood corticosterone and thymus) or ANOVA followed by an LSD test (adrenal glands and spleen). More details in Section 2.1. Data are presented as mean ± SEM overlayed on scatter plots of individual values.

Animals that received corticosterone in drinking water (Figure 1) displayed an increased level of blood corticosterone (Figure 2A) at the time of sample collection, confirming the effectiveness of the treatment. Significant differences were found in case of all treatment durations (5 days of treatment, *p* = 0.01, U = 16, n1 = n2 = 10; 14 days of treatment, *p* = 0.0004, U = 3, n1 = n2 = 10; 28 days of treatment, *p* = 0.0002, U = 0, n1 = n2 = 10) as indicated by the Mann–Whitney *U* test that was applied to data that did not meet the requirement of variance homogeneity. Repeated treatment with corticosterone also decreased the weight of spleen, thymus, and adrenal glands (Figure 2B,C), consistent with expectations based on known effects of glucocorticoids [11,12]. The variance analysis (ANOVA) of adrenal glands showed a significant effect of treatment [F(1,54) = 96.05, *p* < 0.0001] and an insignificant effect of treatment period [F(2,54) = 1.87, *p* = 0.16]. The post hoc test showed that the weight of adrenal glands in corticosterone groups (Figure 2B) was significantly lower compared with the corresponding control groups (*p* < 0.0001 for all treatment durations). The remaining data (spleen and thymus) did not meet the requirement of variance homogeneity and, therefore, were subjected to the Log10 transformation that turned out to be effective only in the case of the spleen data. As a result, the thymic data (Figure 2C) were analyzed with nonparametric Mann–Whitney U test that showed significant differences between corticosterone and control groups for all treatment periods (5 days of treatment, *p* = 0.01, U = 16, n1 = n2 = 10; 14 days of treatment, *p* = 0.0005, U = 4, n1 = n2 = 10; 28 days of treatment, *p* = 0.0002, U = 0, n1 = n2 = 10). Finally, the variance analysis of spleen data after Log10 transformation showed a significant effect of treatment [F(1,54) = 205.44, *p* < 0.0001] and treatment duration [F(2,54) = 11.70, *p* < 0.0001]. The post hoc test showed that the thymic weight in all corticosterone groups (Figure 2D) was significantly lower compared with the corresponding control groups (*p* < 0.0001 for all treatment periods). Furthermore, the decrease was largest in animals receiving corticosterone for 28 days compared with 14 (*p* = 0.0008) and 5 days (*p* < 0.0001) of glucocorticoid treatment, while smaller differences were found between animals receiving corticosterone for 5 and 14 days (*p* = 0.002).

### 2.2. Global Assessment of Hippocampal Transcriptome

#### 2.2.1. Validation of the Dataset

Quality controls are included in the procedure of RNA-seq data processing, including the assessment of the raw data performed separately for each sample (Section 4.5). However, there is a question about the reliability of the entire dataset resulting from multiple steps of data processing. Therefore, we used our entire dataset (Supplementary Data S3) to test predictions based on known functional and anatomical relationships between genes leading to correlated expression [4] including immediate early response genes (*Fos* and *Arc*), hemoglobin genes (*Hba-a1* and *Hbb-bs*), vascular leptomeningeal genes (*Slc13a4* and *Aebp1*) and ribosomal protein genes *Rpl27* and *Fau* (Mendeley Data, doi: 10.17632/dghdbmggc4.4, Supplementary Cluster Files 1. Pearson’s correlation coefficients showed high correlations (0.84 to 0.97, *p* < 0.0001) between genes with close functional relationship (Supplementary Figures S2A–D), low correlation (0.29, *p* = 0.04) between genes with relatively loose functional relationship (hemoglobin and vascular leptomeningeal genes, Supplementary Figure S2F) and insignificant correlations (0.12–0.21, *p* ≥ 0.15) between functionally not related genes (Supplementary Figure S2E,G,H) confirming that the RNA-seq captured expected relationships between genes. Additionally, we have also tested correlations between molecular markers of choroid plexus (*Ttr*, *Folr1*, and *Clic6*) and genes enriched in this brain structure, including *Kl*, *Ecgr4* (*1500015O10Rik*), and *Prlr* (http://mousebrain.org/celltypes/CHOR.html (accessed on 15 July 2024)) [4,13]. Choroid plexus is located in the brain ventricles in the vicinity of the hippocampus and may be inadvertently collected together with hippocampal samples, leading to highly correlated expression of enriched genes [4,13]. The correlations between marker genes were either insignificant (Supplementary Figure S3A,B) or low (0.34, *p* < 0.02, Supplementary Figure S3C) while the correlations between marker genes and enriched genes were mostly insignificant (Supplementary Figure S3D,E,G,H) with exception of *Kl* and *Ecrg4* (Supplementary Figure S3F) that displayed unexpected negative correlation (−0.39, *p* < 0.006). These data indicate negligible contributions of the choroid plexus to the expression of hippocampal genes in the collected samples, confirming the effectiveness of the dissection protocol designed to remove the majority of the choroid plexus [14].

#### 2.2.2. Summary of Hippocampal Transcriptome

**Figure 3:**
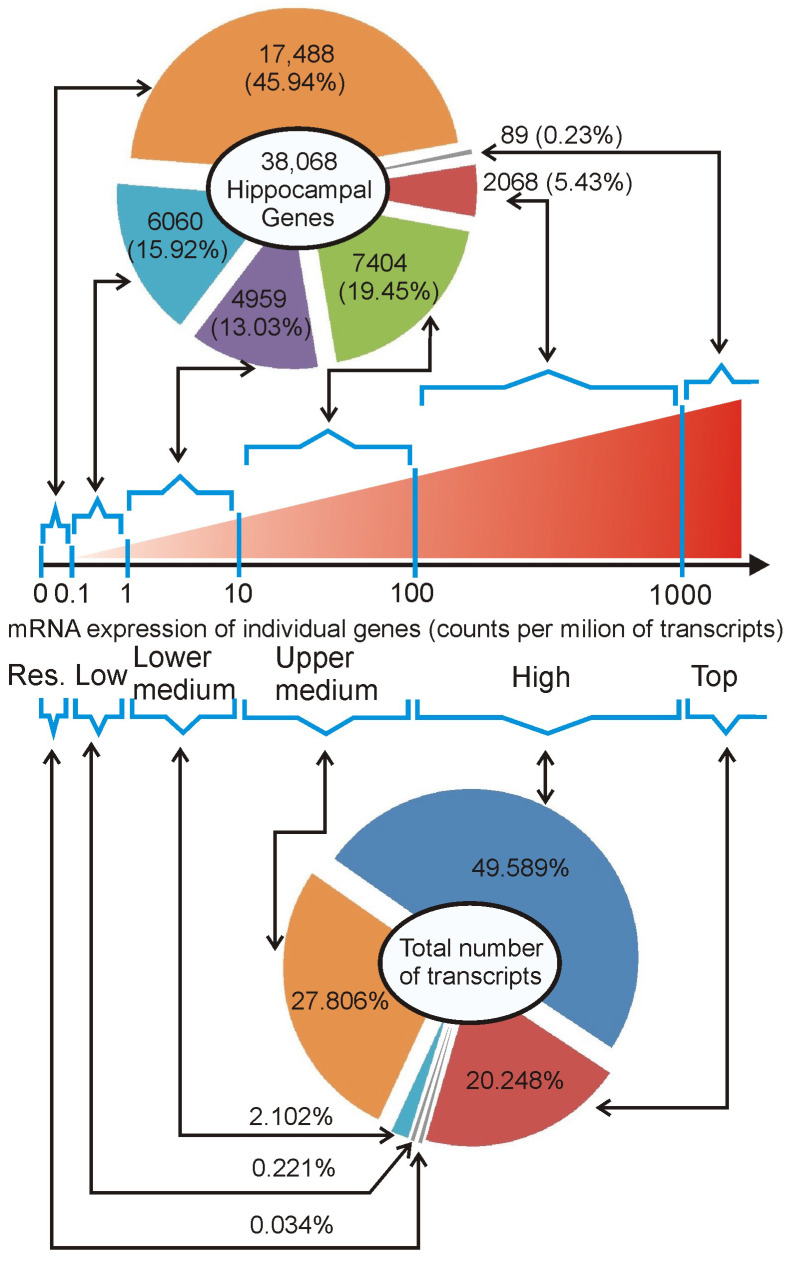
Characteristics of hippocampal transcriptome. All 38,068 genes with detectable expression in at least one sample were grouped into six categories, starting from residual (Res.) to top category based on average expression in all samples. The upper panel shows a number of genes in each category and their contribution to the total number of detected genes. The bottom panel shows the contribution of genes in each expression category to the total number of transcripts (see Section 2.2.2 for more details). The expression level is shown as counts per million (CPM).

The analysis of the RNA sequencing data revealed the expression of 38,087 genes with unique Ensembl IDs that were detected in at least one sample (CPM > 0), while the remaining 15,613 mouse genes were not detected even at the residual level (Supplementary Data S3). The group of detected genes included 38,037 unique gene symbols associated with single Ensembl IDs. In the remaining 50 cases, the gene symbols were associated with more than one Ensembl ID, leading to duplication or triplication of these symbols in the dataset. Several duplicated or triplicated gene symbols have been assigned different gene types in the Ensembl database, and, therefore, they were not integrated into one entry. For example, one copy of the *Bfar* gene is identified as a protein-coding gene while the second copy is identified as a transcribed processed pseudogene. The remaining 18 duplicated or triplicated genes were assigned to the same gene category and, therefore, they were integrated into one entry with summed expression for each sample. These corrections resulted in the final list of 38,068 hippocampal genes that were ordered according to the average expression in all samples (*n* = 48) and grouped into expression categories differing by order of 10 with exception of the two most distant categories including all genes with expression lower than 0.1 CPM and higher than 1000 CPM (Figure 3). The largest number of detected genes (17,488/45.9%) belongs to the lowest expression category (<0.1 CPM) but contributes jointly only 0.03% of all transcripts (Figure 3). In contrast, 89 genes (0.2%) with the highest expression (>1000 CPM) are jointly responsible for 20.2% of transcripts present in hippocampal samples (Figure 3). Furthermore, these 89 top-expressed genes together with 2068 genes with expression between 100 CPM and 1000 CPM contribute jointly 69.8% of all detected transcripts (Figure 3). This shows that the minority of genes (5.6%) are responsible for the majority of transcriptomic activity, while a huge number of detected genes have negligible contributions to total hippocampal transcription.

#### 2.2.3. Determining the Lower Threshold of Biologically Meaningful Expression

The data show that the majority of genes are expressed at low levels. However, the brain is highly heterogeneous in terms of cell types [15] and, therefore, genes that are expressed at high levels but only in a small population of highly specialized cell types may be detected at low levels in homogenized tissue. Therefore, it is necessary to determine a threshold discriminating functionally relevant genes from genes with residual expression, constituting a transcriptomic signal noise. Since our dissection protocol was designed to remove the majority of the choroid plexus [14] consistently with results described in Section 2.2.1, we expect that its marker genes *Ttr*, *Folr1*, and *Clic6* (http://mousebrain.org/celltypes/CHOR.html (accessed on 15 July 2024)) should help us to determine the lower limit of biologically relevant gene expression in hippocampal samples. The average expression of marker genes was within the range of 0.04 to 0.9 CPM (Supplementary Figure S4) and was tentatively assigned to the category of residual (CPM < 0.1) and low (0.1 < CPM < 1) level of expression. In the group of genes with low expression (range between 0.1 and 1 CPM, Supplementary Figure S4) we also found some genes that are present in highly specialized cell types in the hippocampus including *Lao1* (specific for dentate gyrus granule neuroblasts http://mousebrain.org/adolescent/genesearch.html (accessed on 15 July 2024)), *Higd1b* (marker of pericytes http://mousebrain.org/celltypes/PER1.html (accessed on 15 July 2024)) and *Ibsp* (marker of hippocampal trilaminar cells, http://mousebrain.org/celltypes/TEINH13.html (accessed on 15 July 2024)). Therefore, the expression in the range between 0.1 and 1 counts per million [CPM] is still biologically relevant, although it approaches the border between functionally important and residual genes indicated by markers of choroid plexus. This assumption is congruent with the bioinformatic approach used for the selection of biologically meaningful data for the statistical analysis that removed all genes with average expression lower than 0.118 CPM, which is very close to the lower limit (0.01 CPM) of the low expression category (Section 2.2.2). This threshold is further supported by negligible contributions of genes with expression < 0.1 CPM to overall transcriptomic activity (Figure 3).

#### 2.2.4. Characteristics of Expression Categories

The obtained data showed that the median expression for all genes with at least low expression (>0.1 CPM considered as biologically relevant, Section 2.2.3) was 7.8 CPM. Therefore, we termed the expression range between 1 and 10 CPM as a lower medium, while the expression range between 10 and 100 CPM as an upper medium. The remaining two groups are highly expressed genes (the range between 100 and 1000 CPM) and top-expressed (exceeding 1000 CPM) genes, containing a small number of extremely highly expressed genes. To provide a further biological context for the expression categories (Figure 3), we looked at individual genes that are more or less ubiquitously expressed in the brain. We focused on genes involved in common processes such as energy production and signal transduction, hippocampal markers [14], and genes that attracted our attention in the past studies of transcriptomic responses to stress and glucocorticoids [3,4,13,16] with additional support from single-cell transcriptomics of the mouse brain [15] http://mousebrain.org/adolescent/genesearch.html (accessed on 15 July 2024). The top and high expression category contains genes involved in common processes such as energy production, calcium signaling, and excitatory neurotransmission (Supplementary Figure S4). The gene encoding the glucocorticoid receptors and a marker of dorsal hippocampus are expressed within the range of upper medium category, while the lower medium category of expression contains a marker of ventral hippocampus and the well-known immediate early response gene Fos (Supplementary Figure S4). Finally, the low expression category includes markers of several specialized cell types, including dentate gyrus granule neuroblasts and trilaminar cells, while the markers of pericytes are expressed within the range of low and lower medium categories (Supplementary Figure S4).

**Figure 4:**
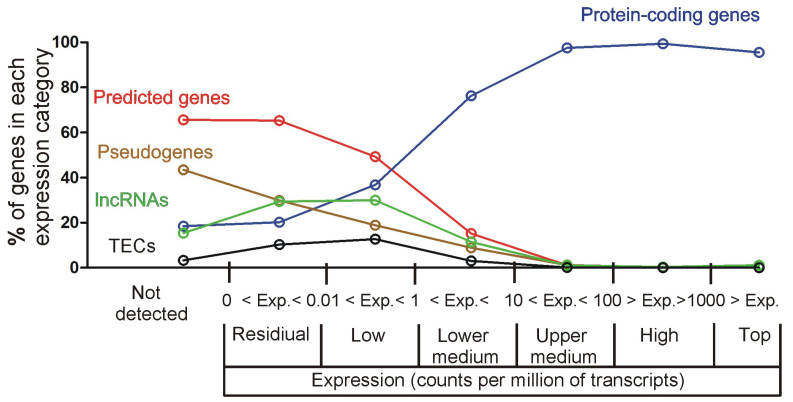
Characteristics of hippocampal transcriptome in terms of the biotypes of detected genes with division into expression categories determined based on mean expression [CPM] in all samples. The undetected group contains genes that are present in the mouse genome (Ensembl database) but were not detected in any hippocampal sample. Information about gene biotypes was retrieved from the Ensembl/Biomart database. Predicted genes constitute a joint category including some genes belonging to various biotypes such as protein-coding, pseudogenes, TECs (To be Experimentally Confirmed), and others. The assignment of genes to the predicted gene category was based on information provided in the Ensembl/Biomart gene description. Exp.—expression.

Additionally, we also looked at information available in the Ensembl/Biomart database, including gene types (defined at www.ensembl.org/info/genome/genebuild/biotypes.html (accessed on 19 February 2023)) and gene descriptions identifying predicted genes (Figure 4). These data showed that the expression categories starting from the lower medium to the top expression level are dominated by genes coding proteins (76.3–95.5%) with a small contribution of predicted genes (15.2 to 0%) while residually expressed genes have an opposite characteristic defined by a large number of predicted genes (65.3%), low number of protein-coding genes (20.2%) and a relatively high number of pseudogenes (29.9%) and lncRNAs (29.4%) (Figure 4). Finally, the low expression category has an intermediate characteristic defined by an increasing number of protein-coding genes (36.8%) and a decreasing number of predicted genes (49.3%) (Figure 4).

### 2.3. Effect of Corticosterone on Hippocampal Transcriptome

#### 2.3.1. General Characteristics of Hippocampal Transcriptome

**Figure 5:**
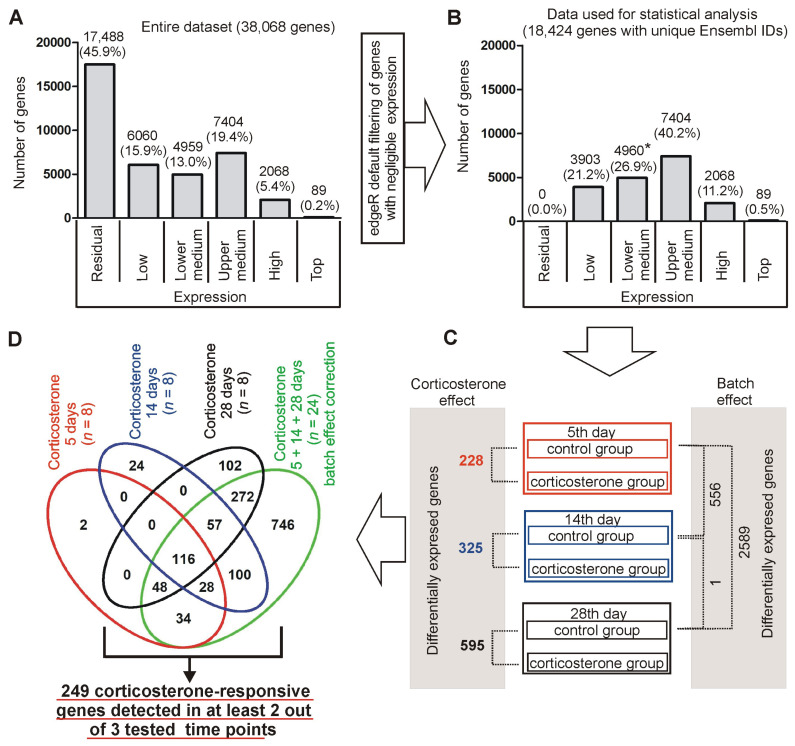
Summary of the analysis of RNA-seq data. (**A**) The number of genes with detectable expression in the analyzed samples. (**B**) The number of genes that passed the edgeR filtering procedure, which removes genes with too low expression to provide biologically and statistically meaningful information. Genes that remained after filtering were subjected to statistical analysis. (**C**) The number of differentially expressed genes in various between-group comparisons with adj *p* < 0.05. (**D**) Venn diagram showing the number of overlapping genes found after different treatment durations and in the pooled dataset with adj *p* < 0.05. * The number of genes in the lower medium category in panel B is higher than in panel A because the statistical analysis was performed on unique Ensembl gene IDs without integration of duplicated gene symbols. As a result, the dataset in (**B**) contains one duplicated gene, *Aldoa*, which is included in both the group of top-expressed genes (ENSMUSG00000030695) and in the lower medium category of expression (ENSMUSG00000114515). In case of the data presented in (**A**), these two Ensembl gene IDs were integrated into one entry, *Aldoa*, and included only in the group of top-expressed genes.

The statistical analysis of the RNA sequencing data was performed on the dataset containing 18,424 genes that passed the filtering procedure (Figure 5B), which was expected to remove genes with too low expression to provide biologically and statistically meaningful information [17,18]. The default filtering settings resulted in the removal of all genes with average expression lower than 0.118 CPM in all samples (*n* = 48). This means that the filtering procedure removed all genes with negligible expression (<0.1 CPM) together with a subset of genes with expression that was close to the lower limit (0.01 CPM) of the low expression category determined previously based on the expression of marker genes (2.2.3).

**Figure 6:**
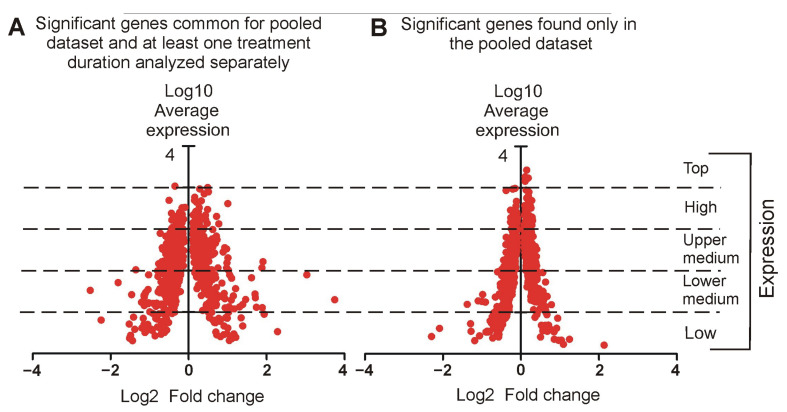
Characteristics of differentially expressed genes (adj *p* < 0.05) identified in the pooled dataset with correction for the batch effect. (**A**) Significant genes common to the pooled dataset and at least one treatment duration that were analyzed separately. (**B**) Significant genes found only in the pooled dataset. Values are log2 fold changes (*n* = 24) plotted against log10 average expression (*n* = 48) calculated for each differentially expressed gene.

Comparison between control and corticosterone-treated animals performed separately for each time point (Figure 5C,D, Supplementary Data S2) revealed a growing number of differentially expressed genes (adj *p* < 0.05), ranging from 228 after 5 days to 595 genes after 28 days of treatment. Nonetheless, a considerable number of genes were common for different treatment durations. In fact, 84.2% (228) of differentially expressed genes after 5 days of treatment were significantly altered in at least one other time point (Figure 5D). Additionally, we have also made a comparison between control groups assigned to various treatment periods and therefore sacrificed at different time points, together with corticosterone-treated animals. Although, the animals assigned to different control groups derived from the same strain, generation and partly from the same litters, they differed in some factors associated with experimental design such as age at the time of sample collection and associated duration of social isolation or any other uncontrollable factor that could affect animals during the prolonged experiment. The statistical analysis revealed differences between control groups, which were the largest in the comparison between 5 and 28 days of treatment (Figure 5C, Supplementary Data S4). Since the experimental design does not allow us to identify a single factor explaining these differences between controls, we will refer to them as a batch effect. These differences between controls, together with a large number of common genes found in corticosterone-treated animals irrespective of the treatment period, prompted us to perform an additional comparison with a correction for the batch effect between pooled data from all corticosterone (*n* = 24) and all control mice (*n* = 24) to increase the statistical power. The analysis of the pooled dataset revealed a much larger number of differentially expressed genes (1401), including most of the significant genes found in at least one treatment period analyzed separately, and 746 new genes (Figure 5D). The comparison between these two subsets (Figure 6) showed that most of the significant genes found only in the pooled dataset (Figure 6B) display low fold changes, although there were also some new genes displaying larger differences but characterized by a low average expression level.

#### 2.3.2. Relationship Between Fold Changes and Average Expression Level

**Figure 7:**
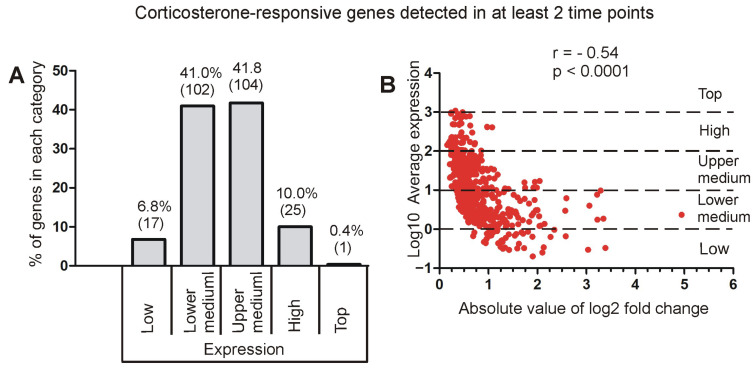
Characteristics of corticosterone-responsive genes displaying significant differences after at least two treatment durations. (**A**) Separation of differentially expressed genes (adj *p* < 0.05) into different categories based on overall expression level. Information about the number of significant genes in each expression category, together with the percentage contribution to the total number of significant genes, is shown over the bars. (**B**) Relationship between expression level and magnitude of responses to corticosterone. Differences between groups (absolute value of log2 fold change) are plotted against overall expression calculated as a log10 average CPM for each treatment period (control + corticosterone group). Points represent differentially expressed genes (adj *p* < 0.05) separately for each treatment duration (*n* = 8). Pearson’s correlation (r), together with the *p*-value, is indicated.

Genes analyzed in homogenized tissue display a large variability in expression levels (Figure 3; Supplementary Figure S4). Therefore, we checked the relationship between the overall expression level and the responsivity to corticosterone. First, we looked at 249 genes that displayed replicable changes in at least two different time points with adj *p* < 0.05 and the same direction of significant responses (Figure 5D, Supplementary Data S2). The separation of these genes into different categories based on overall expression level showed that corticosterone affected the expression mainly in the group of genes with medium expression (lower and upper medium category) that constituted 82.3% of the most replicable findings (Figure 7A). The number of significant genes in different expression categories (Figure 7A) was proportional to the number of analyzed genes (Figure 5B) with two exceptions. First, we found an overrepresentation of genes classified to the lower medium category that constituted 26.9% of all analyzed genes (Figure 5B) but contributed 41% of the most replicable findings (Figure 7A). This indicates that genes with lower medium expression (between 1 and 10 CPM) are the most susceptible to the effects of corticosterone. The second exception was the low expression category, which was clearly underrepresented because it constituted 21.2% of all genes included in the statistical analysis (Figure 5B) but contributed only 6.8% of differentially expressed genes (Figure 7A). Genes with relatively low overall expression levels also displayed large magnitudes of significant changes after treatment with corticosterone, and this relationship was significant as indicated by the correlation between absolute values of log2 fold changes and the log10 mean expression (Figure 7B). A negative correlation between these two parameters indicates that the higher the overall expression level, the smaller the magnitude of changes triggered by the corticosterone (Figure 7B). The same relationship was also observed in the case of significant genes analyzed separately in individual time points and in the pooled dataset (Supplementary Figure S5).

#### 2.3.3. Assessment of Cell Expression Specificity

**Figure 8:**
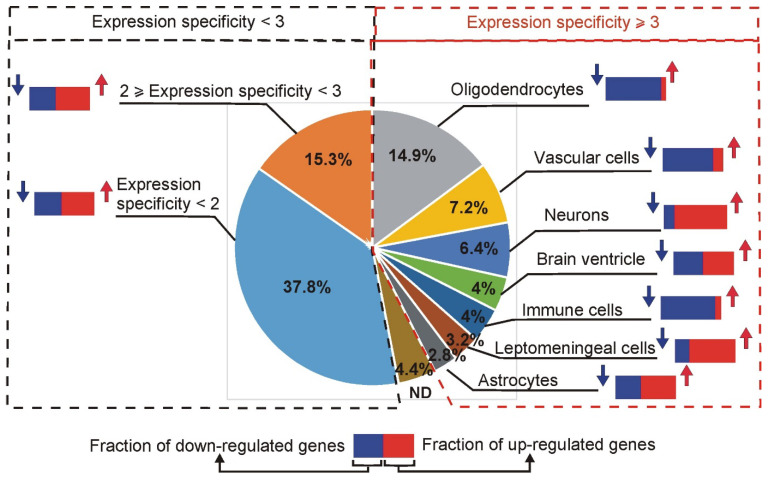
Cell expression specificity of 249 genes responsive to corticosterone that displayed significant differences after at least two different treatment durations. Expression specificity was calculated based on data retrieved from http://mousebrain.org/adolescent/genesearch.html (accessed on 15 July 2024) [15] after selection of cell types that are present in the hippocampus or its direct vicinity (Supplementary Data S1). Expression specificity = 1 means that there are at least two different cell categories with the same level of expression. Expression specificity = 2 and 3 means that the highest expression in a defined cell category is two and three times higher than the expression in any other cell type expected to be present in hippocampal samples, respectively. ND—significant corticosterone-responsive genes that were not present in the mousebrain.org database.

Genes can be expressed ubiquitously in the brain, or their expression can be restricted to some types of cells playing specific functions [15]. To learn more about the typical localization of differentially expressed genes (significant in at least one tested time point), we have retrieved normalized expression values from the atlas of the adolescent mouse brain cells http://mousebrain.org/adolescent/genesearch.html (accessed on 15 July 2024) [15]. This resource provided information about cellular expression of 747 genes responsive to corticosterone, while 36 significant genes (4.6%) were missing in the mousebrain.org database, even after testing synonym gene symbols. Calculated cell expression specificity is provided in Supplementary Data S2. Almost 41% of significant genes displayed low expression specificity that was in the range between 1 (meaning that there are at least two different cell categories with the same level of expression) and 1.99 (meaning that the highest expression in tested cell categories is almost two times higher than expression in any other cell type). The specificity in the range between 2 and 2.99 was found in the case of 14.6% of genes, while the specificity of at least 3 was found in the case of 39.8% of genes. Next, we focused on the group of 249 genes that displayed the most consistent changes in expression, confirmed by significant differences after at least two different treatment durations. Specificity of expression restricted to these genes is shown in Figure 8. Interestingly, the largest group of genes (37/14.9%) with expression specificity ≥ 3 was attributed to oligodendrocytes, and most of them (34) were down-regulated (Figure 8). The second largest group of genes (18/7.2%) with expression specificity ≥ 3 was attributed to vascular cells, and most of them (15) were also down-regulated (Figure 8). Down-regulation was also common in the group of genes attributed to immune cells, while neuronal genes were mostly up-regulated (Figure 8).

#### 2.3.4. Cluster Analysis

**Figure 9:**
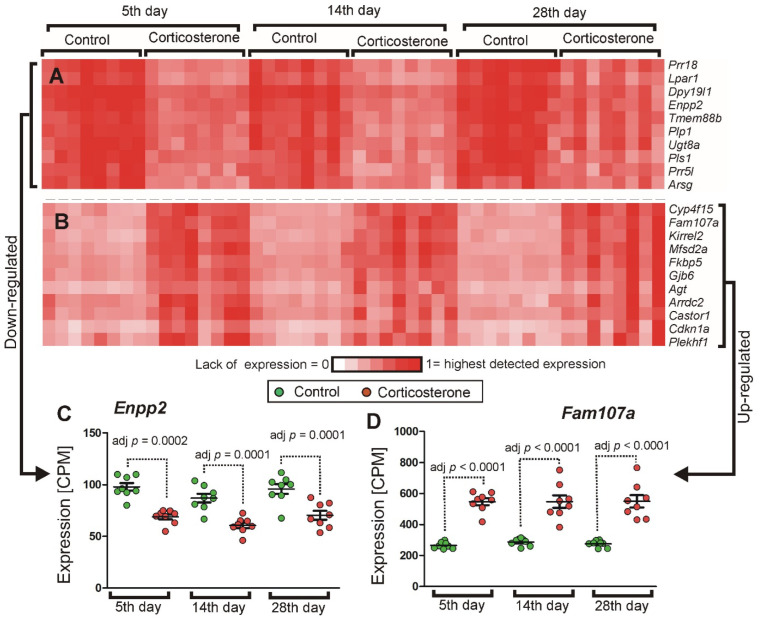
Selected parts of clusters containing genes with highly repeatable responses to corticosterone administered over three different treatment periods. (**A**) Down-regulated genes. (**B**) Up-regulated genes. Examples of genes from panels (**A**,**B**) are shown in panels (**C**,**D**) with mean values and SEM overlayed on scatter plots of individual CPM values from RNA-seq data. Adj *p*-values were determined with edgeR exactTest applied for the RNA-seq data with Bonferroni–Hochberg correction. Complete results of the cluster analysis are provided in Supplementary Data S5 (files cort cluster.cdt, cort cluster.gtr, and cort cluster.jtv for visualization in Java TreeView software) while results of statistical analysis, together with individual CPM values, are available in Supplementary Data S2.

**Figure 10:**
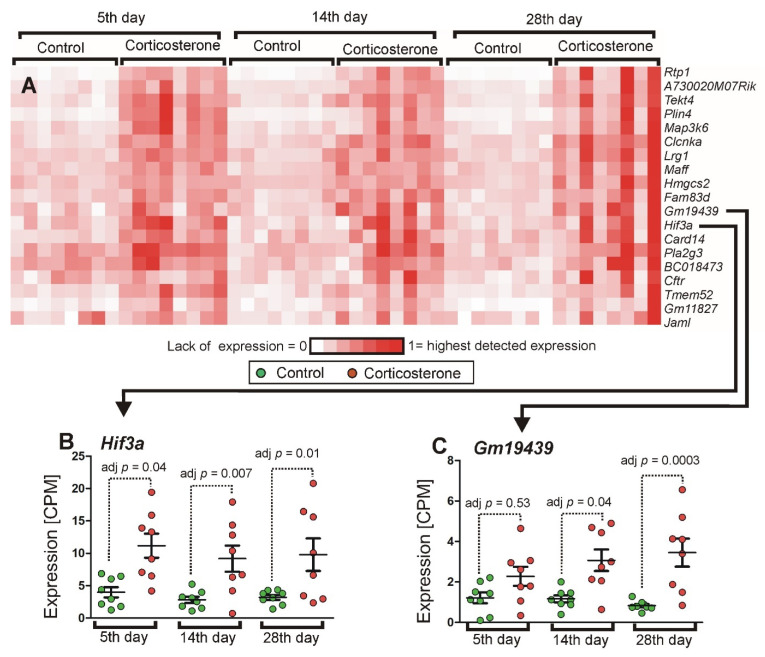
An example of a gene (*Gm19439*) displaying a consistent time-dependent trend in responses to corticosterone, together with other genes sharing similar variability of expression but without consistent time-dependent trends. (**A**) Selected part of the cluster. (**B**) An example of a gene without time-dependent trends. (**C**) An example of a gene displaying a progressive increase in responses to corticosterone. Genes presented in panels (**B**,**C**) are shown as mean values; SEM is overlayed on scatter plots of individual CPM values from RNA-seq data. Adj *p*-values were determined with edgeR exactTest applied for the RNA-seq data with Bonferroni–Hochberg correction. Additional information is provided in the legend for Figure 9.

**Figure 11:**
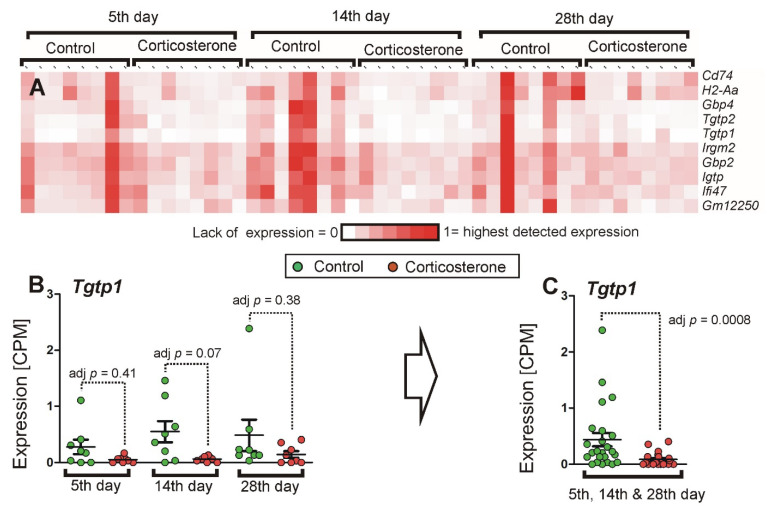
The cluster of genes involved in the immune response that displays a distinct pattern of expression compared with the majority of other down-regulated genes. (**A**) Heat map of gene expression. Most of the genes in this cluster (*Cd74*, *Tgtp2*, *Tgtp1*, *Irgm2*, *Igtp*, *Ifi47*, and *Gm12250*) achieved significance only in the pooled dataset analyzed with correction for the batch effect. An example of such a gene is shown in panel (**B**), presenting separate treatment durations, and panel (**C**) showing pooled data (mean ± SEM overlayed on scatter plots of individual CPM values from RNA-seq data). Adj *p*-values were determined with edgeR exactTest with Bonferroni–Hochberg correction applied for RNA-seq data from individual treatment durations. Pooled data were analyzed with additional correction for batch effect. Additional information is provided in the legend for Figure 9.

The initial assumption was that the progressive duration of glucocorticoid treatment would allow us to differentiate genes into subgroups characterized by discrete patterns of expression resulting from similar biological functions and regulatory mechanisms. We also expected that expression of some genes may either increase or decrease proportionally to the treatment duration due to the accumulation of transcripts or the exaggeration of presumed adverse effects. However, the cluster analysis of 1401 significant genes in the pooled dataset showed a relatively homogenous pattern of expression of the majority of genes that repeated patterns of either up-regulation or down-regulation in consecutive time points without expected time-dependent trends. The homogeneity of expression is shown by the fact that 80.1% of the 776 down-regulated genes displayed a high correlation of at least 0.8, while 63% displayed an even higher correlation of at least 0.92. Similarly, in the case of 625 up-regulated genes, there were 565 (90.4%) genes displaying a correlation of at least 0.79, and 457 (73.1%) that displayed a correlation of 0.9 or higher. The examples of genes with highly repeatable responses to corticosterone are shown in Figure 9, while all clusters are available in Supplementary Data S5 (files cort cluster.cdt, cort cluster.gtr, and cort cluster.jtv) for visualization in Java TreeView software (https://sourceforge.net/projects/jtreeview/files/ (accessed on 9 April 2019) following guidelines provided at https://data.mendeley.com/datasets/dghdbmggc4/4/files/bc38268a-c54b-48bc-85b4-bf0fc52994d2 (accessed on 17 November 2021). Consistent time-dependent trends resulting from progressively changing numbers of transcripts in corticosterone-treated mice were found in the case of very few genes, such as *Gm19439*, *Tlr9*, *Il12rb1*, *Chad*, *Zfp503*, and *Tob2*. These genes were scattered across many subclusters containing genes characterized by similar variability of expression in individual samples but not necessarily similar trends in mean values (Figure 10). Additionally, subclusters characterized by distinct patterns of expression were screened in terms of the biological function of genes, but most of them did not display a clear-cut functional specificity. Furthermore, genes identified as specific for defined types of cells (previous section) were scattered in various subclusters. Clear-cut functional similarities were found only in the case of two subclusters with the most distinct patterns of expression among all down-regulated genes. One of them contains genes coding GTPases involved in immune responses such as *Tgtp1* and *Tgtp2* (T cell specific GTPases), *Igtp* (interferon gamma induced GTPase), *Irgm2* (immunity-related GTPase family M member 2), *Ifi47* (interferon gamma inducible protein 47), *Gbp2* and *Gbp4* (guanylate binding proteins induced by interferon) together with genes associated with histocompatibility complex (*Cd74* and *H2-Aa*) and predicted gene *Gm12250* (Figure 11). The second subcluster contains several genes coding closely related GTPases also involved in immune responses (*Gbp3*, *Gbp5*, *Gbp7*, *Gbp9*), proteins associated with the histocompatibility complex (*H2-K1* and *H2-Q4*), and some other proteins involved in immune response (*Tnfsf10*, *Lgals9*, *Sp100*), together with expressed sequence *AW112010*. However, most of these genes achieved significance only in the pooled dataset and therefore did not contribute to the core part of the hippocampal response to corticosterone, with the exception of single genes per subcluster that achieved significance in at least two separate time points (*Gbp4* and *Gbp5*).

#### 2.3.5. The Effect of Corticosterone on Total Transcriptomic Activity

**Figure 12:**
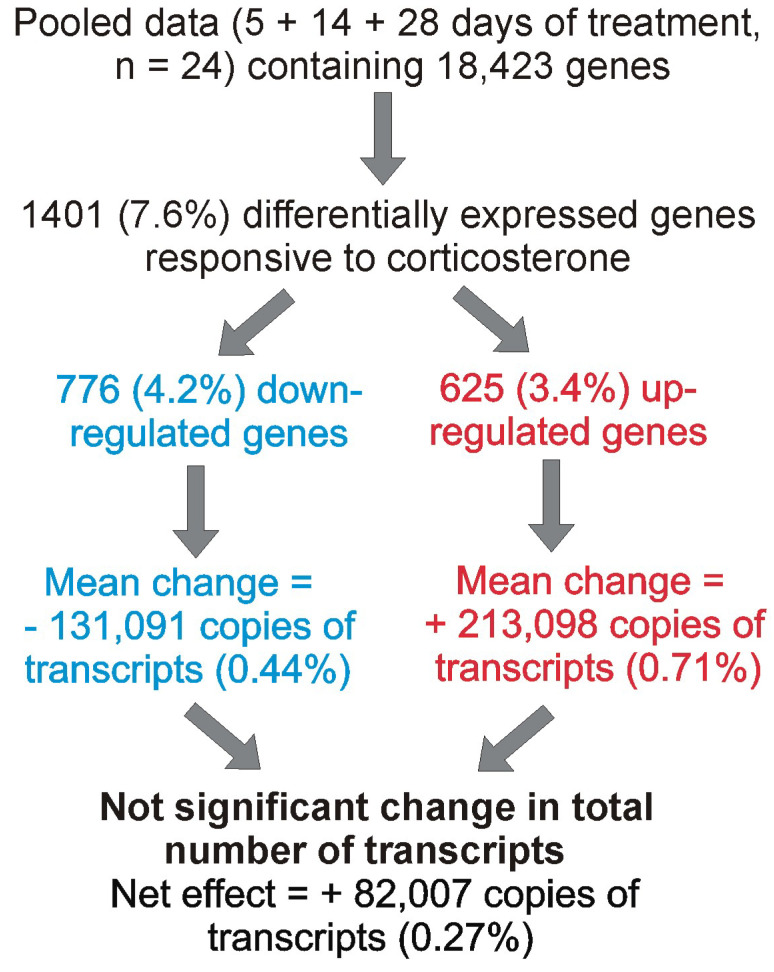
The net effect of corticosterone on the hippocampal transcriptome that was assessed based on differences between the average number of transcripts in control and corticosterone-treated animals, calculated for each differentially expressed gene identified in the pooled dataset.

To assess the upper limit of the effect of corticosterone on the whole transcriptomic activity, we used the dataset with total counts of transcripts for each gene (instead of counts per million) and the results of statistical analysis performed on pooled data (5 + 14 + 28 days) that detected the largest number of differentially expressed genes (1401 genes constituting 7.6% of all analyzed genes; Figure 5D). First, a difference was calculated between the average number of transcripts in control and corticosterone-treated animals separately for each differentially expressed gene. Next, we summed the number of additional transcripts for all up-regulated genes and the number of missing transcripts for all down-regulated genes (Figure 12). This calculation showed that 625 genes up-regulated by corticosterone were jointly responsible for the synthesis of about 200,000 new copies of mRNAs, constituting only 0.71% of the average number of transcripts detected in the control hippocampal samples (Figure 12). Furthermore, this effect was mostly counterbalanced by down-regulated genes, leading to a negligible change in the total number of transcripts that was increased by only 0.27% compared with the control mice (Figure 12). Furthermore, the net effect of corticosterone was not only small but also insignificant as indicated by the statistical analysis (Mann–Whitney U test) performed on the total number of transcripts detected in each sample in consecutive time points (5 days of treatment, *p* = 0.29, U = 22, n1 = n2 = 8; 14 days of treatment, *p* = 0.21, U = 20, n1 = n2 = 8; 28 days of treatment, *p* = 0.53, U = 26, n1 = n2 = 8).

#### 2.3.6. Comparison with Referential Glucocorticoid Datasets

The obtained results were first compared with a referential list of glucocorticoid-responsive genes derived from the meta-analysis of transcriptomic data that were focused mainly on acute effects of glucocorticoids (doi: 10.17632/zy9rbrnrg6.2/ [3]). The referential list was additionally divided into core and extended parts based on the frequency of reporting individual genes (more details in Section 4.9). The analysis of single time points revealed that prolonged treatment with corticosterone induced significant differences in expression of 29 core genes including 12 genes that were significant in every tested time point (*Aldoc*, *Cdkn1a*, *Fkbp5*, *Gjb6*, *Gpd1*, *Klf15*, *Litaf*, *Plekhf1*, *Rhou*, *Sgk1*, *Sult1a1*, *Tsc22d3*), five genes that were significant in two time points (*Klf9*, *Mt2*, *Pdk4*, *Sdc4*, *Wipf3*) and 12 genes (*Arl4d*, *Cables1*, *Ddit4*, *Errfi1*, *Gab1*, *Mertk*, *Nfkbia*, *Nr3c1*, *Pim3*, *Sesn1*, *Sox9*, *Spsb1*) that achieved significance in one tested time point. Finally, an additional 14 core genes (*Gap43*, *Lhfp*, *Bcl6b*, *Arhgef3*, *Rhob*, *Fzd1*, *Azin1*, *Tob2*, *Ehd3*, *Sox2*, *Prr5*, *Sox4*, *Chst1*, *Npy1r*) achieved significance in the pooled dataset analyzed with the correction for the batch effect. Most of these additional genes displayed, however, low magnitudes of changes that were below 20% (log2 ratio < 0.263) compared with the control. Importantly, almost all significant core genes (42 out of 43; exception = *Bcl6b*) displayed a direction of altered expression that was consistent with expectations based on the previous data (Supplementary Data S2). Altogether, these data show that prolonged treatment with corticosterone in mice recapitulated 47.7% of core genes detected mostly after acute treatments with glucocorticoids in various models and species [3]. Smaller consistency was found in the case of an extended list containing 251 genes that were less frequently reported. In total, we found significant differences in expression of 70 (27.9%) genes from the extended list, while the remaining genes were either insignificant in all analyzed data (individual treatment durations and pooled data) or had too low expression to be included in the statistical analysis (6 genes). Consistent findings included 12 genes significant in each time point (*Aif1l*, *Ednrb*, *Endod1*, *Ezr*, *Gpt2*, *Hif3a*, *Idh2*, *Map3k6*, *Mfsd2a*, *Plin4*, *Pltp*, *Slc13a3*), and 10 genes significant at two time points (*Arrdc2*, *Aspa*, *Cryab*, *Cxcr4*, *Fadd*, *Lmcd1*, *Nt5e*, *Pla2g3*, *Snx25*, *Sparc*) while the remaining genes achieved significance either in single time points (24 genes) or in pooled dataset (24 genes). Most of the genes displayed an expected direction of change after corticosterone, with the exception of nine genes that were mainly found in the pooled dataset (6 genes). Altogether, these data indicate that established glucocorticoid genes identified mostly in acute experiments explain the minority of significant genes found after prolonged treatment with corticosterone. For example, 339 genes from the referential list (core and extended) explain that only 15.7% of all 249 genes were found to be significant in at least two tested time points after the prolonged treatment with corticosterone. Therefore, the obtained data were next compared with the list of genes from our previous microarray study that used the same animal model but restricted to one night of treatment with corticosterone [16]. The referential list contained data from three tested time points and included all genes that could be attributed to the total number of 17,444 significant microarray probes without the restriction on fold changes or probe specificities. Therefore, the referential data provided minimal available evidence for changes in gene expression after 12 h of treatment with corticosterone and subsequent 1 to 9 h of rest. In the comparison between the previous and current results (single 12 h vs. repeated 12 h treatments), we have focused on the group of most frequently detected genes that displayed significant differences after at least two different treatment periods (summarized in Figure 5D). The comparison revealed that in the group of 249 most frequently detected genes after repeated treatments, there were 195 genes that were also detected in the morning (1 h of rest) after a single 12 h treatment, and most of them (186/95.4%) displayed the same direction of change. This means that changes in gene expression observed in the morning (1 h of rest) after a single period of overnight treatment [16] explain the expression of 75% (186) of the most frequently detected genes after repeated treatment with corticosterone. Furthermore, responses of an additional 16 genes (6.4%) can be explained by delayed responses with consistent direction of altered expression detected later during the resting period (5th and 9th hour of rest) after a single overnight treatment. A few additional genes common to our present dataset and the previous study displayed an inconsistent direction of change, or consistency could not be unequivocally determined due to the conflicting results obtained from different microarray probes in the previous experiment. In contrast, 32 genes belonging to the group of most frequently detected after repeated treatment were not detected in our previous experiment testing the effect of a single overnight treatment. This indicates that 12.8% of the most frequently detected genes responsive to repeated administration of corticosterone are specific to this treatment regime. Information about other overlapping findings in our previous and current experiments (detected in single time points or only in the pooled dataset) is available in Supplementary Data S2.

#### 2.3.7. Comparison with Referential Stress Datasets

**Figure 13:**
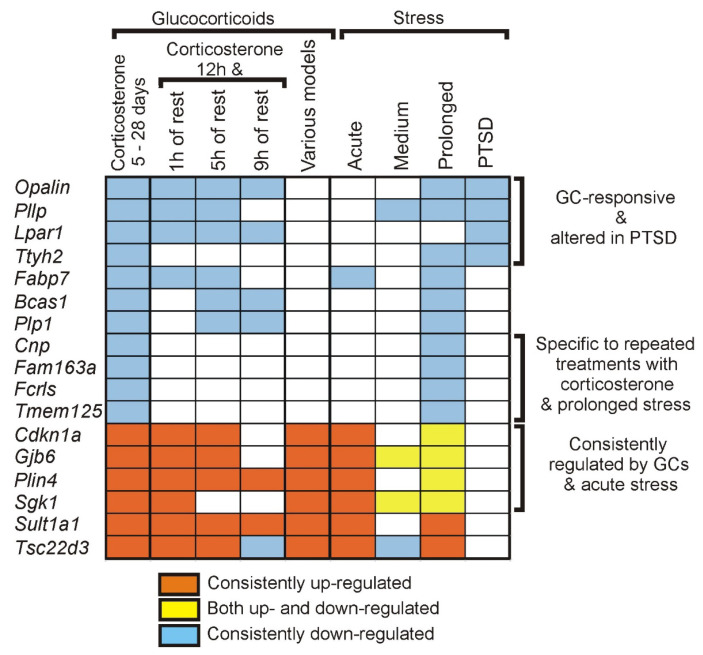
Comparison between present data (corticosterone 5–28 days) and literature data including single overnight (12 h) treatment with corticosterone with different latencies of sample collection [14], most frequently and consistently reported genes in various models testing effects of glucocorticoids [3], transcriptomic responses to stress in animal models [4] and genes associated with human PTSD [4]. The entire dataset is available in Supplementary Data S2.

To learn more about the contribution of corticosterone to the stress response, we have compared transcriptomic responses to corticosterone (present experiment) with changes in gene expression induced by stress [4]. The referential list of stress-responsive genes (Supplementary Data S2) is based on the previous meta-analysis of experiments performed mostly in mice and rats [4] and contains genes responsive to stress of various durations (acute, medium, prolonged), stress in general and genes found to change expression in brains of patients suffering from post-traumatic stress disorder (PTSD). The comparison (Supplementary Data S2) revealed that in the group of all 1529 genes responsive to repeated administration of corticosterone in our experiment (all significant genes from individual time points and pooled dataset), there are 332 genes (21.7%) that are significantly overrepresented in studies testing the effect of stress on gene expression in various species and brain areas. The overlap between corticosterone and stress datasets (Supplementary Data S2) was highest in case of genes that achieved significance after all three tested treatment periods (40.5%/49 genes) and gradually decreased in group of genes significant in two time points (32.3%/43 genes), one time point (20.8%/111 genes) and genes that achieved significance only in the pooled dataset (17.6%/131 genes). Some stress-responsive genes display a preference for either increased or decreased expression, as indicated in available data from the literature [4]. Therefore, we have also looked at the consistency in the direction of altered expression in stress and corticosterone datasets, focusing on the group of the most frequently detected genes after repeated treatment with corticosterone (significant after at least two treatment durations). The criterion for assuming preferential expression was 75% of experiments indicating the same direction of response to stress. This threshold was achieved by 26 genes responsive to prolonged stress (lasting more than 1 week) that were also altered by corticosterone after at least two different treatment durations, and most of them (25 genes) displayed the same direction of responses to glucocorticoids and stress. Similarly, consistent direction of responses to stress and corticosterone was found in the case of the majority of genes (24 out of 25) that displayed a preferential direction of responses to acute stress and were significantly regulated after at least two treatment durations (Supplementary Data S2, Figure 13). An expected direction of altered expression was also found in the case of four genes (*Opalin*, *Pllp*, *Ttyh2*, *Lpar1*) responsive to repeated treatment with corticosterone and exclusively down-regulated in patients with PTSD (Supplementary Data S2, Figure 13). Finally, we have found consistent changes in the expression of genes *Cnp*, *Fam163a*, *Fcrls*, and *Tmem125* that are specific to prolonged stress and repeated treatment with corticosterone (Supplementary Data S2, Figure 13).

#### 2.3.8. qPCR Validation

RNA sequencing data were validated with the qPCR method that was used to quantify expression of exemplary genes, including four down-regulated genes (*Opalin*, *Aplnr*, *Fabp7*, *Bfsp2*) and six up-regulated genes (*Tekt4*, *Fmo2*, *Agt*, *Etnppl*, *Sult1a1*, *Lrg1*). All these genes displayed significant differences in RNA-seq data after all treatment durations, with the exception of *Bfsp2*, which achieved significance after 14 and 28 days of treatment, while approaching significance (*p* = 0.07) after 5 days of treatment. The obtained PCR results showed a significant correlation with RNA-seq data (*p* ≤ 0.0004) for all tested genes (Supplementary Figures S6–S8). The correlation ranged from 0.94 in the case of *Etnppl* (Supplementary Figure S6A) to 0.51 in the case of *Lrg1* (Supplementary Figure S8C). The average correlation for all validated genes was 0.77 and was similar to the correlation between reference gene PCR Cq values and RNA concentration (r = 0.76; Supplementary Figure S1), which can be considered as a joint measure of sampling, transcription, and measurement errors occurring during multiple steps of PCR analysis and RNA-seq. A high correlation between RNA-seq and PCR data also resulted in congruent differences between groups for most of the validated genes (9 out of 10). In case of congruent genes, the significant differences were confirmed by PCR in 24 out of 27 comparisons between groups (9 genes x 3 time points) while minor deviations were found in remaining three comparisons including two cases when differences only approached significance in PCR (*p* = 0.07 and 0.08, Supplementary Figure S7B,F) in contrast to RNA-seq and one opposite case with significant differences in PCR (Supplementary Figure S7D) while the corrected *p*-value in RNA-seq data only approached significance (*p* = 0.07, Supplementary Data S2). The single incongruent gene was *Lrg1* that had the lowest correlation between RNA-seq and PCR data (r = 0.5, Supplementary Figure S8C) and displayed highly insignificant PCR differences after two out of three treatment periods (Supplementary Figure S8D) in contrast to RNA-seq data that showed significant differences after all treatment durations (Supplementary Data S2). This gene also had the lowest concentration in the retrotranscribed pool of samples, as indicated by the highest Cq values and the largest number of individual PCR tests that failed to detect the gene in 40 (27.8%) out of 144 wells with hippocampal samples (48 samples run in triplicate). This indicates that the concentration of the *Lrg1* gene approached the detection threshold, increasing the likelihood of sampling errors that compromise the PCR reaction [19,20].

### 2.4. Western Blot

**Figure 14:**
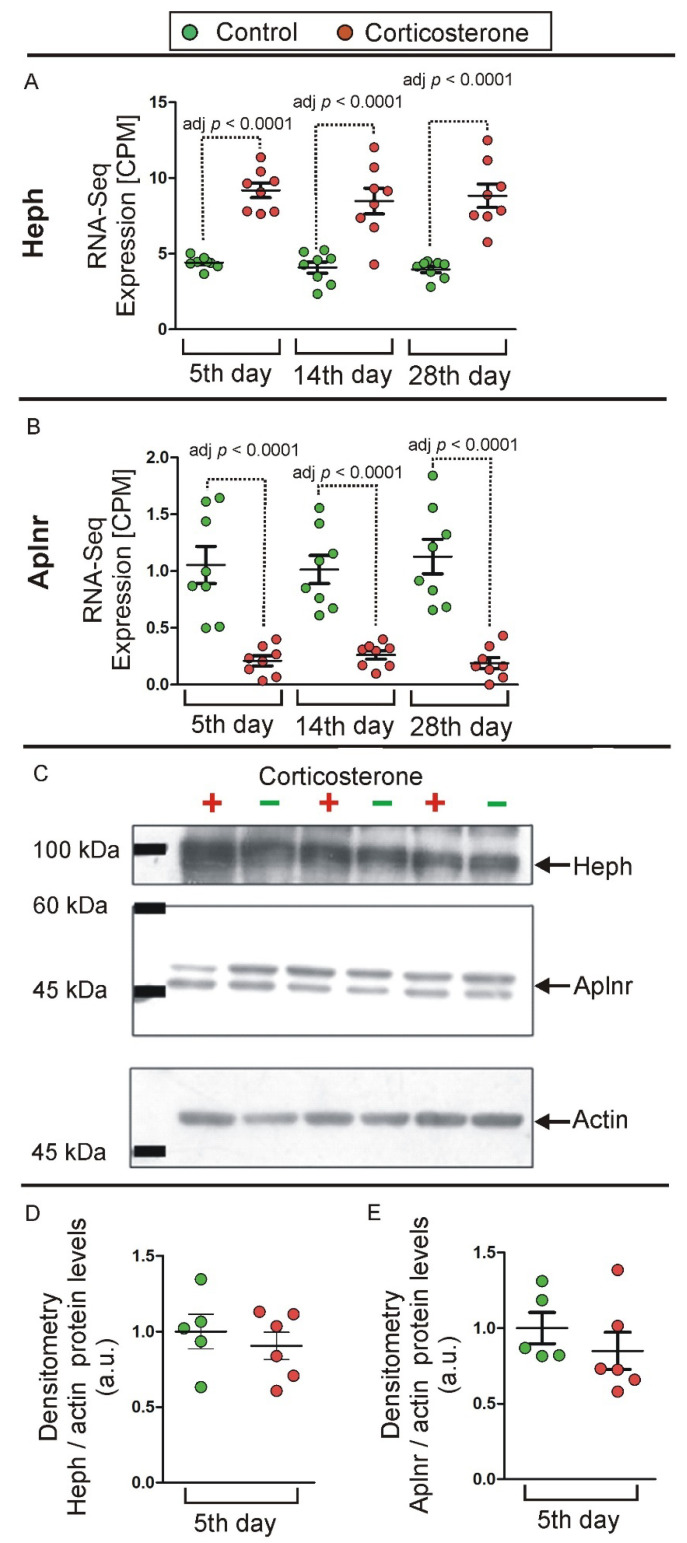
Comparison of mRNA levels detected by RNA-seq (**A**,**B**) and protein levels detected by Western blotting method (**C**), together with representative Western blot densitometry analyses (**D**,**E**). Reactive bands were quantified relative to those of actin using a Molecular Imager with Quantity One software 4.6.8 (Bio-Rad, Hercules, CA, USA). Data are presented as mean ± SEM overlayed on scatter plots of individual values. Adj *p*-values were determined with edgeR exactTest applied for the RNA-seq data with Bonferroni–Hochberg correction.

A separate group of control (*n* = 5) and corticosterone (*n* = 6) mice underwent a 5-day treatment and were then used for the preliminary protein level assessment of hippocampal samples. Samples were divided into two batches containing both control and corticosterone-treated mice and were analyzed using the Western blotting method. Two Western blottings were performed for each batch, but neither of them showed differences in expression of Heph and Aplnr proteins, despite highly reproducible differences in mRNA levels detected by RNA-seq (Figure 14). The lack of differences between groups in Western blotting was confirmed for both proteins by analysis of variance (Heph: F(1,9) = 0.43, *p* = 0.53; Aplnr: F(1,9) = 0.84, *p* = 0.38).

## 3. Discussion

The large experiment with 48 animals and three parallel treatment regimens enabled us to identify replicable transcriptomic responses to prolonged treatment with corticosterone that significantly altered the expression of 116 genes after all treatment durations and 133 genes after two treatment durations, with a consistent pattern of either up- or down-regulation in independent comparisons (Supplementary Data S2). This allows us to narrow the focus on the group of 249 most replicable findings out of 783 genes found to be significantly altered after at least one treatment duration, and an even larger group of 1401 genes found significant in the pooled dataset (Figure 5D, Supplementary Data S2). Large sample sizes that increase statistical power, together with replicability assessment, are important because they solve the problem of large signal noise that is common in transcriptomic studies performed typically on a small number of analyzed samples [3,4].

Interestingly, our most replicable findings (249 genes) contain only 6.8% of well-established genes that are responsive to glucocorticoids, mostly after acute treatments, and the overlap can be increased to 15.7% after inclusion of an additional 251 genes that were less frequently detected in the past experiments [3]. This means that the well-established genes explain only a minority of our current findings. Importantly, any individual study included in the previous analysis of literature data [3] tested the effects of glucocorticoids dissolved in drinking water and administered during a natural period of spontaneous activity that is associated with circadian changes in gene expression [21,22]. Therefore, to learn more about the effects specific to treatment with corticosterone entrained to the sleep–wake cycle, we have compared our current results with the data from the previous study that used the same experimental setup but with treatment restricted to one night and expression detected with microarrays instead of RNA-seq [16]. This comparison showed that effects observed after a single overnight treatment with corticosterone [16] explain 81% of the most consistently detected genes after repeated treatments, including not only significant differences but also a consistent direction of altered expression. This approach allows, therefore, the identification of genes that are responsive to overnight treatment and replay of a similar pattern of expression during subsequent circadian cycles of elevated levels of corticosterone (Supplementary Data S2). Finally, the comparison between datasets revealed a unique set of genes in our current data that change expression only in response to repeated treatments with corticosterone and, therefore, may underlie chronic effects of glucocorticoids. The comparison between present and previous data [16] indicates that such genes constitute 12.8% of the most replicable findings after repeated treatments (Supplementary Data S2) including genes with highly specific brain expression localized in neurons (*Epha10*, *Calb1*, *Glt8d2*), vascular system (*Aplnr*, *Gbp4* and *Ushbp1*) and oligodendrocytes (*Efhd1*, Gm13293, Phldb1, *Fgfr2*). Importantly, the group of genes specific to repeated treatments with corticosterone contains genes that are also specific to the prolonged stress (*Cnp*, *Fam163a*, *Fcrls*, *Tmem125*) or prolonged stress/human PTSD (*Ttyh2*) and display a consistent direction of change (Figure 13). Two of these genes are specifically expressed in oligodendrocytes (*Cnp* and *Tmem125)* while *Fcrls* is highly specific for the category of the brain immune cells, including microglia and perivascular macrophages (Supplementary Data S2, http://mousebrain.org/adolescent/genesearch.html (accessed on 15 July 2024)). Although the function of these genes is not well understood, the localization of expression indicates that some unique effects of repeated treatment with corticosterone and prolonged stress may affect oligodendrocytes and immune brain cells. It should also be noted that genes specific to oligodendrocytes are common among all 249 genes displaying replicable responses to corticosterone and constitute the largest group of genes with cell-type-specific expression (Figure 8). Less common are genes with specific expression in vascular cells, neurons, astrocytes, immune cells, and cells associated with brain ventricles (choroid plexus and ependymal cells). Such a large number of oligodendrocyte-specific genes explains the increased sensitivity of white matter to glucocorticoids compared with grey matter [23]. The data also indicate that glucocorticoids may affect gene expression in all components of the central nervous system, consistent with data showing expression of glucocorticoid receptors both in neuronal and non-neuronal cell types [24].

An important question associated with glucocorticoid research is the similarities and differences between the pharmacological effects of glucocorticoids and the effects of endogenous glucocorticoids released during a stress response, together with other mediators of the stress response. Our comparison between the present transcriptional data and the referential list of stress-response genes [4] showed that common genes for the stress and glucocorticoid response constitute 36.9% of the most replicable transcriptomic responses to repeated treatment with corticosterone (92 out of 249 genes). This comparison with the referential stress dataset is limited, however, by the fact that a large number of genes are underrepresented in published data, which means that they are reported less frequently than expected by chance in multiple stress experiments due to various technical and methodological reasons [4]. Therefore, the referential list of genes that are overrepresented in multiple stress studies [4] is biased for genes that were identified during early stages of genome research and had distinct sequences facilitating the design of selective microarray probes. This means that the estimated overlap provides information about a minimal number of common genes for the corticosterone and the stress response, while the upper limit of similarities cannot be estimated with currently available data. Nonetheless, the already identified patterns of gene expression that are common for corticosterone and stress responses constitute an important advancement in deciphering molecular mechanisms from transcriptomic data.

Another approach that can be used to gain functional insight into transcriptomic mechanisms is the identification of genes sharing distinct patterns of expression during treatments differing in duration or intensity [4,13,25]. Therefore, our initial assumption was that the experiment with three different treatment periods would lead to differentiation of transcriptomic response into groups of genes with discrete patterns of expression resulting from similar biological functions and regulatory mechanisms. Furthermore, we expected that genes would display growing magnitudes of altered expression reflecting the growing duration of treatment. However, the majority of regulated genes did not display clear-cut time-dependent trends caused by either progressive accumulation or decline in transcripts over time. Instead, the dominant pattern was repetition of similar magnitudes of changes in expression over time (Figure 9, Supplementary Data S5), indicating that homeostatic processes were sufficient to prevent the buildup of mRNAs during consecutive days and weeks of treatment entrained to the sleep–wake cycle. Furthermore, most of the differentially expressed genes displayed a similar pattern of expression in individual samples as indicated by high correlations in two major clusters grouping down and up-regulated genes (Figure 9, Supplementary Data S5). An exception was the two most distinct subclusters among down-regulated genes. The subclusters composed of *Cd74*, *H2-Aa*, *Gbp4*, *Tgtp2*, *Tgtp1*, *Irgm*2, *Gbp2*, *Igtp*, *Ifi47*, *Gm12250* and *Gbp5*, *Tnfsf10*, *Gbp9*, *Gbp7*, *Gbp3*, *H2-Q4*, *H2-K1*, *AW112010*, *Lgals9*, *Sp100* (Figure 11, Supplementary Data S5) contain genes involved in immune responses including numerous GTPases and are expressed in the brain vascular system (http://mousebrain.org/adolescent/genesearch.html (accessed on 15 July 2024)). At least part of these results is relevant for the stress response because the cluster containing down-regulated genes *Gbp4*, *Ifi47*, *Irgm2*, *Igtp*, and *Tgtp1* was previously identified after chronic stress in mice [13].

The final issue considered in our study was the interpretation of changes in gene expression in the context of the entire hippocampal transcriptome. Obtained data showed that the largest changes in expression of corticosterone-responsive genes are restricted to genes that have relatively low expression levels (Figure 7, Supplementary Figure S5) and a small contribution to the overall pool of mRNAs in the hippocampus (Figure 12). This means that even a large change in terms of the number of affected genes leads to a small change in the number of mRNA copies. For example, 625 up-regulated genes contribute jointly about 200,000 new copies of mRNAs that constitute only 0.71% of the total pool of hippocampal mRNAs, and this effect is counterbalanced by down-regulated genes, leading to negligible net increases in the number of transcripts by 0.27% (Figure 12). Changes smaller than 1% of the total number of mRNAs indicate that the transcriptomic changes induced by corticosterone exert low-cost effects on the brain. Such low-cost transcriptomic effects have an obvious survival advantage because they help to conserve energy and have a simple mechanistic explanation. It is because synthesis of mRNA is a slow process constituting a bottle neck in gene expression from DNA to proteins [26]. Therefore, cells can synthesize only a limited number of mRNA copies for each gene, and the same number of new copies makes a large difference in case of genes with a low basal number of mRNA copies, but has a negligible or very small effect in case of genes with a high basal number of mRNA. This negative relationship between effectiveness in gene regulation and the average number of gene copies in hippocampal tissue is shown in Figure 7 and Supplementary Figure S5. This also means that proteins for which there are many copies of mRNA can be effectively regulated during a short period of time only at the post-transcriptional stage of gene expression. Consideration of these facts helps to understand discrepancies between transcriptomic and proteomic effects of glucocorticoids and provides a rationale for changing only the levels of mRNAs without concomitant changes in the level of proteins (Figure 14). Survival in the environment involves many different and frequently opposite scenarios, including an increased or decreased activity (fighting or escaping versus resting and hiding), hyperthermia or hypothermia, food, water, or oxygen deprivation, and various injuries that may or may not occur. Initiating or stopping the most time-consuming processes, such as transcription, will help the organism to prepare in advance for various scenarios associated with the fight-or-flight reaction. In such a situation, the change in the level of proteins is expected to require additional signals triggered by real emergencies, leading to protein synthesis or degradation. Although such a regulatory mechanism of corticosterone signaling is only a hypothesis, the translation on demand is already known to regulate some cellular processes [26,27]. It should also be noted that the average lifetime of proteins in the mouse brain is 9 days, although the half-lives may range from 2 h to even 1 year [28], indicating that some proteins have not only a slow degradation rate but also restricted synthesis under basal conditions. Levels of such proteins are expected to be resistant to changes in the amount of mRNA, unless there is a sufficient amount of time for protein synthesis or there is a rapid protein degradation triggered by disturbed homeostasis. In advance preparation of mRNAs for various scenarios with subsequent on demand synthesis or degradation of protein is advantages not only because the synthesis of mRNA is a bottleneck in gene expression [26] but also because it consumes a relatively low amount of energy constituting only 10% of the energetic expenditures required for protein synthesis [27]. Preparation of mRNAs for various scenarios with subsequent on-demand changes in protein levels is consistent with many observations indicating that glucocorticoids induce context-specific effects in the brain [29] and constitute a basis for understanding variability in side effects of glucocorticoids [30]. This mechanism also helps to understand the observation that some tissue-specific effects of glucocorticoids seem to rely on single genes despite a large number of regulated genes [31]. Finally, the limitations of regulatory mechanisms that rely upon the basal level of mRNAs lead to the conclusion that obtaining the full picture of mechanisms underlying the effects of glucocorticoids depends on the integration of transcriptomic and proteomic data.

## 4. Materials and Methods

### 4.1. Animals

Male mice (Swiss-Webster) used in the experiment were obtained from the breeding colony located at the Institute of Genetics and Animal Biotechnology (Jastrzebiec, Poland). The experiments were performed on 71 mice, including 60 animals used in the RNA-seq experiment with 10 mice per group (Figure 1) and 11 mice (*n* = 5 and 6) used for preliminary analysis of brain proteins. The initial number of 10 animals per group in the main RNA-seq experiment was larger than planned number of sequenced samples (*n* = 8 per group) as a precaution in case of any loss of animals during prolonged experiment, loss of samples during dissection or tissue processing and failure to obtain sufficient quantity or quality of RNA in some samples (sample selection is described in Section 4.4). The animals were 3.5 months old, weighed 35.7 ± 1.3 g (mean ± SEM) and were group housed (4–5 mice per cage) in cages with fine sawdust bedding under a standard light cycle (12/12 h), temperature (22 ± 2 °C) and humidity (55 ± 5%) with free access to dry food (Labofeed H, Kcynia, Poland) and tap water. The environment was enriched with cardboard tubes and paper towels, providing material for nest building. The experiment was performed with the permission of the Second Local Ethical Committee in Warsaw (permit number: WAW2/090/2018) in accordance with the Polish Act of 15 January 2015 on the protection of animals used for scientific and educational purposes.

### 4.2. Pharmacological RNA-Seq Experiment

The pharmacological experiment was preceded by a 3-week habituation procedure that was used in our previous stress [13,25] and corticosterone studies [16]. The habituation procedure started with the separation of mice into individual cages that were moved from the breeding colony to the experimental room, which was dedicated only to this experiment to limit the human activity that could disturb the animals. Single-housed animals were next left undisturbed for three weeks to habituate them to the new conditions. The separation of animals into individual cages solves the problem of stress associated with antagonistic behaviors between dominant and subordinate male mice [32,33] and enables the selection of individual mice for tissue collection without disturbing other animals during the final stage of the experiment. The length of the habituation period is based on observations of changes in reactivity to environmental stimuli [25] and corticosterone levels [34]. The pharmacological treatment started after the completion of the habituation phase. The procedure was the same as in our previous experiment [16] with the exception of treatment durations. The mice were assigned to three corticosterone-treated groups (*n* = 10) that received the glucocorticoid dissolved in drinking water (100 µg/mL) for 5, 14, or 28 nights and to three control groups (Figure 1). Administration of drugs in drinking water is a noninvasive method allowing for stress-free treatment during natural sleep–wake cycles. Limiting stress associated with experimental procedures is especially important in the case of studies testing glucocorticoids because endogenously released stress hormones constitute a confounding factor obscuring the effects of experimental treatments. The dose of corticosterone was based on literature data and our previous experiments [11,16,35,36,37]. Available data [16,38] and our current experiment show that this dose increases blood corticosterone to physiological levels observed after stress [39,40,41,42].

The corticosterone-treated groups and corresponding control groups contained siblings from about five different litters to enable the comparison between brothers. Because of hydrophobic properties, the corticosterone (Sigma-Aldrich, St. Louis, MO, USA) was initially dissolved in a 30% solution of cyclic oligosaccharide hydroxypropyl-cyclodextrin (Sigma-Aldrich) with the help of a vortex and magnetic stirring bar and was next diluted to obtain the final concentration of corticosterone (100 µg/mL) and hydroxypropyl-cyclodextrin (0.45%) following previously described procedure [16,43]. Control animals received drinking water only with the addition of hydroxypropyl-cyclodextrin (0.45%). Bottles with corticosterone solution (22 mL) or vehicle (22 mL) were changed every evening at the end of the light phase to provide fresh solutions before the start of the nocturnal activity of mice. Euthanasia (cervical dissociation) and sample collection were performed during the first hour of the light phase following the last night of treatment (Figure 1). Cages with animals intended for sample collection were quietly transferred one by one from the experimental room to the adjacent dissection room immediately before the euthanasia. Control and corticosterone-treated animals were sacrificed in alternating order. Entire-hippocampi dissection was performed according to the procedure developed in our laboratory and described in detail by Jaszczyk et al. [14] in a methodological paper supported with video. Collected hippocampi were placed in freezing vials, frozen in liquid nitrogen, and stored in a refrigerator at −80 °C. Additionally, we also collected trunk blood for corticosterone measurement and internal organs (thymus, spleen, and adrenal glands) to verify the long-term effects of corticosterone, which is known to decrease the weight of these organs [11,12]. The organs were weighed immediately after dissection.

### 4.3. Analysis of Blood Samples

Blood was collected in Eppendorf tubes containing 20 µL of 0.4 mM Na2EDTA and centrifuged for 10 min at 5000 RPM and +4 °C to obtain plasma that was next stored at -20 °C. The plasma corticosterone level was measured by an enzyme-linked immunosorbent assay with a wide detection range (0–2250 ng/mL, Demeditec Corticosterone rat/mouse ELISA kit DEV9922, Kiel, Germany) according to the manufacturer’s protocol https://www.demeditec.com/en/products/corticosterone-rat-mouse-elisa-dev9922/dev9922-corticosterone-ratmouse-elisa-170608-e.pdf (accessed on 11 October 2019) with two technical replicates for each sample. The absorbance was read at 450 nm.

### 4.4. Sample Preparation and RNA Sequencing (RNA-Seq)

Total RNA was extracted from 60 individual hippocampal samples (10 in each group, Figure 1) using the GeneMATRIX Universal RNA Purification Kit (Eurx, Gdańsk, Poland) according to the manufacturer-supplied protocol. The RNA samples obtained were additionally purified with the DNase Max kit (Qiagen) to remove remnants of DNA. The quantity and quality of RNA samples were assessed by spectrophotometry (ND-1000, Nanodrop) and microcapillary electrophoresis (Bioanalyzer 2000, Thermo Fisher Scientific, Waltham, MA, USA). Eight samples from each group with the highest RNA quality (260/280~2.1, RIN > 9) were next selected for the RNA sequencing that was outsourced to Polgen (Lodz, Poland) and performed by BGI (BGI Tech Solutions, Hong Kong) on the DNBSEQ G400 platform. About 1.9 µg of RNA from each sample was sent for sequencing, while the remaining RNA was used for the PCR validation. The sequencing was performed on stranded libraries and the protocol included mRNA enrichment with Oligo dT, RNA fragmentation and reverse transcription (second-strand cDNA synthesis with dUTP instead of dTTP for preparation of stranded specific mRNA libraries), end repair, A-tailing, adaptor ligation, PCR amplification, single strand separation and cyclization, DNA nanoball synthesis and sequencing on DNBseq G400 platform (BGI Tech Solutions, Hong Kong). The sequencing depth was 30 M while the read length was 100 bp. Raw RNA-seq data were deposited in the NCBI Gene Expression Omnibus (GEO) database (accession number GSE280140).

### 4.5. Analysis of RNA-Seq Data

The first steps of the sequencing data analysis were automated using the RASflow workflow [44] run inside a Singularity container [45]. These steps included: (a) quality validation of raw and trimmed data in fastq format using MultiQC [46] and FastQC tools (https://www.bioinformatics.babraham.ac.uk/projects/fastqc/ (accessed on 23 July 2021); (b) trimming of adapters using Trim Galore (https://www.bioinformatics.babraham.ac.uk/projects/trim_galore/ (accessed on 30 August 2021)); (c) alignment of reads to mouse GRCm39 genome assembly using hisat2 [47]; (d) quality validation of the aligned data using MultiQC and BamQC (https://github.com/s-andrews/BamQC (accessed on 30 August 2021)); (e) feature counting using featureCounts [48]. The count files generated in the final step of the RASflow workflow were analyzed with the edgeR package (https://bioconductor.org/packages/edgeR (accessed on 30 August 2021) [49]). Mapped reads were expressed as counts per million (CPM), which are scaled by the total number of reads. The data derived from all 48 samples were filtered by the filterByExpr function [50] with the min.count parameter set to 10, following the edgeR guidelines [18,50]. This filtering procedure restricted the statistical analysis to genes that met the requirement of expression level ≥ 0.33 counts per million (CPM) in at least 8 samples (defined as the smallest group size in the analyzed experiment) out of all 48 samples. The cut-off threshold (0.33 CPM) was calculated based on a formula included in the filterByExpr function ((min.count parameter/median library size) × 1,000,000). The data were normalized with the normLibSizes function using the default TMM method [18]. Differences between groups were tested with the edgeR exactTest separately for each time point. Pooled data (all corticosterone samples vs. control samples) were analyzed with correction for the batch effects available in the edgeR package. The Bonferroni–Hochberg method [51] was used to calculate adjusted *p*-values. Fold changes were log2-transformed.

### 4.6. Assessment of Cell Expression Specificity

Genes can be expressed ubiquitously in the brain, or their expression can be restricted to some types of cells playing specific functions [15]. To learn more about the expression specificity of genes responsive to corticosterone, we have retrieved normalized expression values from an atlas of the adolescent mouse brain cells http://mousebrain.org/adolescent/genesearch.html (accessed on 10 July 2023) [15].

The mousebrain.org database was searched using official gene symbols from our dataset (783 genes significant in at least one tested time point) or with gene synonyms when the currently used symbol was absent in the database. Retrieved data were restricted to cells specific to the hippocampus and dentate gyrus or cells ubiquitously localized in the brain including neurons, astrocytes, oligodendrocytes, ependymal cells, vascular endothelial cells, vascular smooth muscle cells, leptomeningeal cells, pericytes, perivascular macrophages, microglia, and choroid plexus epithelial cells, following the cell taxonomy available at http://mousebrain.org/taxonomy/r1_neurons.html (accessed on 28 January 2024). We have also created additional categories encompassing excitatory neurons, dentate gyrus granule neurons, immature neural cells, inhibitory neurons, immune cells (microglia and perivascular macrophages), vascular cells (pericytes, vascular endothelial and smooth muscle cells) and brain ventricle system cells (ependymal and choroid plexus epithelial cells) based on biological function and similarity in gene expression. All cell types and cell categories are provided in Supplementary Data S1. The cell expression specificity was calculated as a ratio between the highest expression in a defined category and the highest expression level found in cells from the remaining categories. For example, gene *Fcrls* has the highest normalized expression (=2.21) in MGL1 microglia, belonging to the category of brain immune cells encompassing microglia and perivascular macrophages. The highest expression level is divided by the highest expression found in cells from all other categories (in this case, 0.0141 detected in ABC vascular leptomeningeal cells) to calculate the expression specificity. The obtained value 156.7 indicates that the highest expression found in brain immune cells is 156.7 times higher than the highest expression detected in any other non-immune cells that can be encountered in hippocampal samples. This procedure was repeated for all cell categories, and the highest specificity score was selected for each gene. In a few cases, we encountered a problem of indefinite values resulting from dividing by 0. In these cases, we substituted 0 with 0.0001 to calculate the cell expression specificity following the approach used previously [4].

### 4.7. Cluster Analysis

The cluster analysis was performed to identify groups of genes sharing similar patterns of expression that are expected to result from overlapping functions or anatomical and cellular distributions [4,13,52]. Clustering was performed on all genes that displayed significant differences in the pooled dataset. The results of the cluster analysis performed directly on CPM values could not be visualized effectively because of very large differences between genes in the basal level of expression, preventing the setting of a contrast threshold that could show differences between samples for all analysis genes. Therefore, the data were transformed by calculating the ratio between each individual CPM value and the largest CPM value separately for each gene. As a result, the expression of all genes is in the range between 0 and 1, with 1 indicating the highest detected expression in the analyzed samples. Transformed data were clustered hierarchically using the complete linkage method and uncentered correlation as a similarity metric [52,53]. Clustering was performed with the Cluster 3.0 program (http://bonsai.hgc.jp/~mdehoon/software/cluster/software.htm (accessed on 27 March 2019), and the resulting data were visualized in Java Treeview software version 1.1.6r4 (https://sourceforge.net/projects/jtreeview/files/ (accessed on 9 April 2019)) [54].

### 4.8. Real-Time qPCR Validation of RNA-Seq Data

Ten selected genes (*Agt*, *Aplnr*, *Bfsp2*, *Etnppl*, *Fabp7*, *Fmo2*, *Lrg1*, *Opalin*, *Sult1a1*, *Tekt4*) were analyzed by Real-Time qPCR using mRNA that remained after RNA sequencing. Primers were designed using the Primer-BLAST tool [55]. The designed primers were located on two different exons and contained all transcript variants of each gene. The annealing temperature for each primer was determined by a three-step amplification process using PCR with a temperature gradient in the range of 55 °C to 65 °C. The relative expression of selected genes was calculated using the expression of the *Gapdh* reference gene, which achieved the highest correlation between qPCR Cq values (number of amplification cycles needed to reach the quantification threshold) and RNA concentration (Supplementary Figure S1). This gene also displayed the highest expression (indicated by the lowest Cq values) compared with three other reference genes (*Hmbs*, *Ywhaz*, and *Tbp*). Therefore, in principle, *Gapdh* is expected to be the least susceptible to sampling errors during PCR analysis [19,20]. The primer specifications, including reference genes, can be found in Table 1. RNA samples were extracted using the GeneMATRIX Universal RNA Purification Kit (EURx Ltd., Gdansk, Poland). The quantity and quality of RNA samples were assessed by spectrophotometry (ND-1000, Nanodrop, Waltham, MA, USA). Retro-transcription to cDNA was performed with 500 ng of total RNA from each sample using the First Strand cDNA Synthesis Kit (Roche, Basel, Switzerland). Real-Time qPCR was performed in a Light Cycler 96 (Roche) with FastStart Essential DNA Green Master kit (Roche) according to the manufacturer’s protocol, and each gene was tested in triplicate on three separate plates. Each plate contained three negative controls without cDNA. A series of five-fold dilutions of the total cDNA was used to assess PCR efficiency. The reaction volume was set at 20 µL. The melting curve analysis of PCR products was performed with software dedicated to the Light Cycler 96 (Roche) to confirm amplification specificity. Relative expression of validated genes was calculated using the Pfaffl method [56].

### 4.9. Comparison with Referential Datasets

The obtained data were compared with other transcriptomic datasets relevant for brain response to glucocorticoids and stress. The list of well-established genes responsive to glucocorticoids (doi: 10.17632/zy9rbrnrg6.2) was derived from the previous meta-analysis [3] based on 17 in vivo and in vitro studies [57,58,59,60,61,62,63,64,65,66,67,68,69,70,71,72,73] that most frequently tested acute effects of glucocorticoids (1 to 6 h). The referential list included most frequently and consistently reported genes that were subdivided into core part (88 genes that displayed the same direction of change in at least four studies) and extended part (251 genes that displayed the same direction of change in three studies) following our previous studies [4,16]. These two datasets constitute 0.9% (core dataset) and 2.6% (extended dataset) of all reported genes displaying significant differences after treatment with glucocorticoids [3]. Five gene symbols that were not found in our present data were updated to the current official symbol using Ensembl/Biomart genome browser (www.ensembl.org) with the following settings: Mouse genes (GRCm39) selected as a dataset, gene synonym selected in filters, and gene name selected in attributes. The human symbol GYG1 was changed into the current mouse symbol using the human genome GRCh38.p14, and the mouse gene name selected from the attributes/homologues/orthologues/mouse orthologues command. The preferential direction of change was indicated as up-regulation in case of genes that were reported at least three times more frequently to increase expression after glucocorticoids, while down-regulation was indicated in case of genes that were reported at least three times more frequently to decrease expression.

The list of genes responsive to corticosterone after a single overnight administration in drinking water was derived from our previous study [16]. The annotation of all significant probes was repeated using updated Ensembl/Biomart database for mouse reference genome GRCm39 with Agilent microarray SurePrint G3 GE 8x60K V2 platform selected in filter settings, Ensembl database for mouse strain genomes with SurePrint G3 GE 8x60K platform selected in filter settings and producer database (Catalog Gene Lists/SurePrint G3 Mouse GE v2 8x60K Microarray available at https://earray.chem.agilent.com/earray/ (accessed on 2 February 2024) used to annotate microarray probes missing in the Ensembl.

The list of genes responsive to stress was derived from the recent meta-analysis of 82 transcriptomic studies [4]. The list contained five categories: genes responsive to acute (restricted to 1 day), medium, and prolonged (>1 week) stress, human genes altered in post-traumatic stress disorder, and a joint category of stress-responsive genes. The categories of genes responsive to stress contain genes that were reported more frequently than expected from random effects in the entire stress dataset or its subparts restricted to stress of various durations [3]. Therefore, it should be noted that the joint category of stress-responsive genes is not a compilation of genes responsive to acute, medium, and prolonged stress, and some genes were found significantly overrepresented only in the entire dataset or its subparts. Gene nomenclature was changed for some genes since the time of this meta-analysis. Therefore, the obsolete gene symbols were updated to the current official symbol using Ensembl/Biomart genome browser with the following settings: mouse genes (GRCm39) selected as a dataset, gene synonym selected in filters, and gene name selected in attributes. Genes that increased expression in at least 75% of experiments were labeled as preferentially up-regulated, while genes that decreased expression in at least 75% of experiments were labeled as preferentially down-regulated.

### 4.10. Western Blot

Genes chosen for preliminary protein assessment were *Heph* and *Aplnr*. The genes were selected based on several reasons, including reproducible expression changes in our experiment (Supplementary Data S2), functional interest, and discrete localization of expression in the brain ventricular (*Heph*) and vascular (*Aplnr*) systems. Additionally, the selection of these genes was supported by changes in expression, which were also reported in some other glucocorticoid datasets [16,74,75,76,77]. Western blot analysis was performed on single hippocampi dissected from the left or right hemisphere in alternating order. To obtain total cellular extracts, the whole hippocampus was homogenized in RIPA buffer (10 mM Tris pH8, 150 mM NaCl, 1 mM EDTA, 1% Igepal (NP40), 0.1% SDS) supplemented with a protease inhibitor cocktail (Sigma-Aldrich, Burlington, MA, USA) and phenylmethanesulfonylfluoride (PMSF; Sigma-Aldrich). The homogenate was left on ice for 30 min and then centrifuged (6000× *g*, 15 min, 4 °C). Protein concentrations were determined by Bradford assay (Bio-Rad, Hercules, CA, USA), and total protein extracts (20 µg of protein) were subjected to SDS-PAGE electrophoresis. The samples were boiled for 10 min before loading. Before membrane blocking with 5% skim milk, transfer efficiency was confirmed by Ponceau-S staining. The list of primary and secondary antibodies used is given in Table 2. After incubation with primary antibody (overnight, 4 °C), blots were washed (TTBS buffer) and incubated with horseradish peroxidase-conjugated secondary antibodies at room temperature for 1 h, followed by visualization with the enhanced luminescence kit (Thermo Fisher Scientific, Waltham, MA, USA). For the quantitative analysis of protein content, reactive bands were quantified relative to those of actin using a Molecular Imager with Quantity One software 4.6.8 (Bio-Rad).

### 4.11. Statistics

This section contains a description of statistical methods applied to all data, with the exception of the analysis of RNA sequencing data, which is described in Section 4.5. The obtained data were first tested using C Cochran, Hartley, Bartlett, and Levene’s tests to verify variance homogeneity. Data that did not meet the requirement of variance homogeneity were subjected to Log_10_ and square root transformations [78] and were tested again with the C Cochran, Hartley, Bartlett, and Levene’s tests. Logarithmic transformation is more efficient in correcting variance inhomogeneity and, therefore, was used as the first-choice approach. However, data that could not be corrected with logarithms due to the presence of values equal to zero (blood corticosterone) were subjected only to square root transformation. The significance of differences between groups was tested either with variance analysis followed by Fisher’s least significance difference (LSD) test (applied for data with homogenous variances) or with the nonparametric Mann–Whitney U test that was used for data that did not meet this requirement even after the transformation. Correlations between data were tested using Pearson’s test. The statistical analysis was performed with the Statistica software, release 7.1. Values are presented as scatter plots with mean ± SEM.

## Figures and Tables

**Table 1:** PCR primers.

Gene Name	Forward or Reverse Primer Sequence		Annealing Temperature	Efficiency
*Agt*	F	GCGGAGGCAAATCTGAACAAC	60 °C	100.6%
	R	CTCGTAGATGGCGAACAGGA		
*Aplnr*	F	TCGTGGTGCTTGTAGTGACC	60 °C	91.5%
	R	ATGCAGGTGCAGTACGGAAA		
*Bfsp2*	F	TGCTGCCCTCAGTGTAGAGTTA	60 °C	115.8%
	R	GGCGTCGTGCAAGCTGTTTT		
*Etnppl*	F	TTGGTGAAGGACCGTGAGAAA	60 °C	94.9%
	R	AACTTTGCATCGTCTTCCGTG		
*Fabp7*	F	GGTGGCAAAGTGGTGATCC	60 °C	92.6%
	R	ATCCCCAAAGGTAAGAGTCACG		
*Fmo2*	F	AGGCTCCATCTTCCCAACCG	60 °C	94.3%
	R	AAGGCGAGTTCATCCAGGTAGT		
*Lrg1*	F	GTCTCTTGGCAGCATCAAGG	60 °C	107.6%
	R	GAATTCCACCGACAGATGGAC		
*Opalin*	F	AGACTGTGGTCCCTCTATTGG	60 °C	86.6%
	R	GGGTTCATTTCATGTGTGGGTG		
*Sult1a1*	F	GATGGGAAAGTGTCCTATGGGT	60 °C	92.9%
	R	TGAAGGATGTGTGGTGAACAATTA		
*Tekt4*	F	GCCTTTACCAGCGGTCACA	60 °C	71.2%
	R	CTGTTGGTCTTGACTGCGATG		
*Gapdh*	F	TCAAGCTCATTTCCTGGTATGACAA	60 °C	99.8%
	R	TCTCTTGCTCAGTGTCCTTGCT		
*Ywhaz*	F	TTGAGCAGAAGACGGAAGGT	60 °C	96.9%
	R	GAAGCATTGGGGATCAAGAA		
*Tbp*	F	GCAGTGCCCAGCATCACTATT	60 °C	108.5%
	R	AAGCCCTGAGCATAAGGTGG		
*Hmbs*	F	TCCTGGCTTTACTATTGGAG	60 °C	87.4%
	R	TGAATTCCAGGTGGGGGAAC		

**Table 2:** List of antibodies used in Western blot analysis.

Target Protein	Primary Ab	Dilution	Secondary Ab	Dilution	
**Apelin receptor** (Invitrogen, Waltham, MA, USA, Catalog # PA5-114830, RRID:AB_2899466)	Affinity-purified rabbit anti-recombinant protein corresponding to the human APLNR (Accession P35414), amino acid residues A147-W197 https://www.thermofisher.com/order/genome-database/dataSheetPdf?producttype=antibody&productsubtype=antibody_primary&productId=PA5-114830&version=Local (accessed on 28 May 2024)	1:1000	Goat anti-rabbit IgG (whole molecule), polyclonal, peroxidase conjugate, affinity isolated antibody (Sigma-Aldrich Catalog # A6154, RRID: AB_258284) https://www.sigmaaldrich.com/PL/pl/product/sigma/a6154#product-documentation (accessed on 28 May 2024)	1:20,000	
**Hephaestin** (Affinity Biosciences, Cincinnati, OH, USA, Catalog # DF13057, RRID:AB_2846018)	Affinity-purified rabbit anti-human recombinant protein, corresponding to a region within the internal amino acids https://www.affbiotech.com/download/pdf/DF13057 (accessed on 28 May 2024)	1:1000	Goat anti-rabbit IgG (whole molecule), polyclonal, peroxidase conjugate, affinity isolated antibody (Sigma-Aldrich Catalog # A6154, RRID: AB_258284) https://www.sigmaaldrich.com/PL/pl/product/sigma/a6154#product-documentation (accessed on 28 May 2024)	1:20,000	
**Actin** (ACTN05 (C4) Thermo Fisher Scientific, Cat# MA5-11869, RRID:AB_11004139)	Rabbit monoclonal, anti-chicken gizzard actin https://www.thermofisher.com/order/genome-database/dataSheetPdf?producttype=antibody&productsubtype=antibody_primary&productId=MA5-11869&version=Local (accessed on 28 May 2024)	1:1000	Goat anti-rabbit IgG (whole molecule), polyclonal, peroxidase conjugate, affinity isolated antibody (Sigma-Aldrich Catalog # A6154, RRID: AB_258284) https://www.sigmaaldrich.com/PL/pl/product/sigma/a6154#product-documentation (accessed on 28 May 2024)	1:20,000	

## Data Availability

All data are provided in the manuscript and external repositories. Raw RNA-seq data were deposited in the NCBI Gene Expression Omnibus (GEO) database (accession number GSE280140). Supplementary files with processed data are available at https://data.mendeley.com/datasets/w3df8dhfwb/3 (DOI:10.17632/w3df8dhfwb.3) (accessed on 14 May 2025).

## Supplementary Materials

The following supporting information can be downloaded at https://data.mendeley.com/datasets/w3df8dhfwb/3 (accessed on 14 May 2025).

## Abbreviations

The following abbreviations are used in this manuscript:

CPM	Counts Per Million
lncRNA	Long non-coding RNA
TEC	To be Experimentally Confirmed
